# Application of Microalgal Stress Responses in Industrial Microalgal Production Systems

**DOI:** 10.3390/md20010030

**Published:** 2021-12-26

**Authors:** Jia Wang, Yuxin Wang, Yijian Wu, Yuwei Fan, Changliang Zhu, Xiaodan Fu, Yawen Chu, Feng Chen, Han Sun, Haijin Mou

**Affiliations:** 1 College of Food Science and Engineering, Ocean University of China, Qingdao 266003, China; wj7154@stu.ouc.edu.cn (J.W.); wangyuxin7553@stu.ouc.edu.cn (Y.W.); fyw@stu.ouc.edu.cn (Y.F.); zhuchangliang@ouc.edu.cn (C.Z.); 2 School of Foreign Languages, Lianyungang Technical College, Lianyungang 222000, China; wuyj5131@163.com; 3 State Key Laboratory of Food Science and Technology, Nanchang University, Nanchang 330047, China; luna_9303@163.com; 4 Heze Zonghoo Jianyuan Biotech Co., Ltd, Heze 274000, China; sunhanias@163.com; 5 Institute for Advanced Study, Shenzhen University, Shenzhen 518060, China; sfchen@szu.edu.cn

**Keywords:** adaptive laboratory evolution, microalgal production, environmental tolerance

## Abstract

Adaptive laboratory evolution (ALE) has been widely utilized as a tool for developing new biological and phenotypic functions to explore strain improvement for microalgal production. Specifically, ALE has been utilized to evolve strains to better adapt to defined conditions. It has become a new solution to improve the performance of strains in microalgae biotechnology. This review mainly summarizes the key results from recent microalgal ALE studies in industrial production. ALE designed for improving cell growth rate, product yield, environmental tolerance and wastewater treatment is discussed to exploit microalgae in various applications. Further development of ALE is proposed, to provide theoretical support for producing the high value-added products from microalgal production.

## 1. Introduction

**Figure 1:**
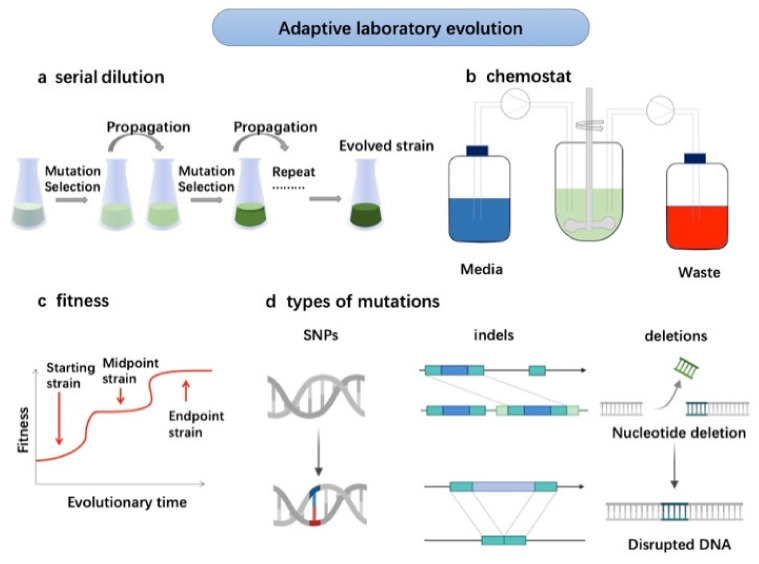
Adaptive laboratory evolution for strains improvement. The serial dilution is applied for mutation selection during the propagation until the evolved strains obtained (**a**). The improved strains can be cultured for high-density cultivation and wastewater treatment (**b**). The strains undergo starting, midpoint and endpoint periods (**c**) with types of nucleotide deletion and disrupted DNA (**d**).

Adaptive laboratory evolution (ALE) refers to obtaining the expected biological evolution under given laboratory conditions. As an innovative method, it is to make up for the neglect of molecular genetic mechanisms in Darwin’s theory of evolution and its development. As an experimental method, it uses high-throughput sequencing of DNA as a tool to effectively simulate the evolutionary process of the selection (Figure 1).

Compared with natural selection, ALE is the process of implementing the "rules" of natural evolution for specific populations (mainly microorganisms) in the laboratory under controlled conditions, exerting pressure on them to obtain the required characteristics until the new strains with favorable mutations are developed [1]. Over the past few decades, ALE has been successfully used to develop microorganisms with the required phenotypes.

Microalgae have attracted extensive attention from all walks of life and penetrated various fields. For example, they are used for the production of bioactive compounds [2] and bioenergy [3,4], as well as in wastewater treatment facilities [5]. Therefore, microalgae are being intensively developed and utilized for various applications. To better utilize and control microalgal biomass and product yield, microalgae are suitable for ALE research with their advantages of fast growth rate, short generation time, easy to control in different cultivation systems and convenience preservation. Altering the environment for microalgal culture through ALE, we can use low-cost investment in exchange for the higher biomass concentration and product yield.

ALE is less common in microalgal experiments at the early stage. Recently, numerous studies explored the effects of environment conditions or chemical compounds on cell growth and product accumulation; the genetic materials are not changed in a short time [6]. The microalgal production still needs condition configurations for high productivity in each batch cultivation. However, ALE induces the accumulation of beneficial mutant genes, resulting in new genotypes more suitable for the stressful environment [7]. Later experiments revealed the adaptation mechanism of planktonic alga, *Skeletonema costatuni*, under strong light and a high temperature, which provided a foundation for ALE in microalgal engineering [8].

The improvement of microalgal strains is one of the major applications of ALE in microalgae (Figure 1). It can exert pressure on the process of microalgae growth and metabolism through batch or continuous culture, to make microalgae constantly adapt to the new environment, and evolve towards the beneficial mutation, including high biomass concentration and product yield. The serial dilution is applied to obtain the evolved strain, which usually continues for 3 months to 2 years. The fitness of strain undergoes starting, midpoint and endpoint periods. Then, the improved strains can be cultured in chemostat for high-cell density growth or wastewater treatment. Unlike genetic engineering, ALE does not need to know the genetic basis of the target phenotype in advance [9], the generated mutants are generally recognized as safe (GRAS) [10]. ALE with the above advantages, has been initially developed mainly for bacterial and fungal models, allowing cells containing beneficial random gene mutations to reproduce more rapidly under environmental stress. When this strategy is applied to microalgae, it is mainly used to improve the growth rate, product yield, stress tolerance and the ability of nitrogen and phosphorus removal in wastewater [7]. These can meet the industrial demand of microalgal production.

## 2. Adaptive Laboratory Evolution Experimental Design 

In an ALE experiment, strains are cultured in a unique pressure under artificial environmental conditions for a long time. Therefore, the formation of evolved strains is promoted, and populations best adapted to the growth environment outperform the residual ones [11]. It is necessary for ALE to take various factors into consideration simultaneously in microalgae, such as the strain, stressful condition, cultivation condition and cultivation strategy. 

### 2.1. Cultivation Modes

A range of culture methods have been successfully used for ALE, including continuous culture, batch culture and staged culture.

#### 2.1.1. Continuous Culture

The continuous culture can maintain process conditions with constant nutrient supply and cell densities [11]. Similar to “Bioreactor Batch Cultivations”, the same bioreactor method can be used for continuous evolution experiments. The process of this training is that the addition of fresh nutrients into the medium during exponential growth at a suitable rate would allow the biomass to increase at a given rate indefinitely. A steady state can be achieved so that the microbial population grows at a constant rate in a constant environment [12]. However, there are also some disadvantages, such as the high expense and difficult control.

#### 2.1.2. Batch and Fed-Batch Culture

Batch culture refers to a method of culturing strains using a certain amount of medium in a closed reactor. The characteristic of this mode is to load culture medium and inoculate bacteria at the beginning of culture. The volume of culture medium and culture temperature during the process are maintained.

Fed-batch culture is a variation on batch culture, which is fed continuously or sequentially with substrate without removing any of the biomass. Compared with conventional batch culture, fed-batch culture has several advantages, including sufficient nutrients, decreased fermentation time and higher productivity [13]. However, the operation of the fed-batch culture is more complicated, which requires an appropriate feeding strategy in detail. Erythritol production by fed-batch culture of *Trichosporon* sp. resulted in a high constant productivity [14]. According to the general expression patterns, there were outgoing differences in gene expression profiles between the batch and fed-batch cultures that can be attributed to the fed-batch process [15]. Fed-batch culture has been applied widely to increase biomass and lipid productivity [16].

#### 2.1.3. Staged Cultivation

Conditions for product accumulation are usually different from those for biomass accumulation [17], the strategy of two-stage process has been found to conquer this paradox. According to this method, there is a new ALE approach named as “chemical modulators based adaptive laboratory evolution” (CM-ALE). The first step used acetyl-CoA carboxylase (ACCase), as a pressure to increase the lipid and docosahexaenoic acid (DHA) productivity of strains by 50% and 90%, respectively. Then, the second step used a sesamol based on the stress of ACCase, to increase the cell growth rate and make the productivity of lipid and DHA up to 100% and 130%, respectively [18]. This demonstrated that the two-step CM-ALE can achieve mutual improvement between desired products and cell growth. The two-stage process was considered as the better approach for productivity improvement. However, the choice of pressure is indispensable with the strategy applied [19]. The understanding of the relationship between carbon metabolism and the ROS quenching mechanism can provide strategies for microalgal production [18].

In addition, heterotrophic cultivation has become a tempting option to increase cell density by overcoming microalgal dependency on light [20]. Therefore, ALE strategy domesticating autotrophic strains to heterotrophic types is a promising approach for high density cultivation of microalgae.

### 2.2. Choice of Stress Conditions and Equipment

During microbial ALE, a strain is cultivated under clearly defined conditions for prolonged periods of time. The selective stress serves as the foremost step for the success of ALE, which can be classified into environment stress and the nutrient stress [21]. Microbial characteristics should be considered for selecting pressure. The special properties of microalgal strains are anticipated to amplify to improve the productivity in industrial production. For example, *Chlorella vulgaris*, *Neochloris oleeoabundans* and *Scenedesmus obliquus* [22] are known as producing lipids, so pressure promoting lipid accumulation is preferentially selected, such as nitrogen starvation and high light. 

The culture equipment is another important factor. The approaches for ALE usually include serial transfer, colony transfer or chemostat culture [17]. The chemostat is commonly used for the continuous addition of medium and simultaneous removal of culture broth [23]. In addition, ALE is suitable for automation in photobioreactor (PBR) to achieve continuous monitoring, improve experimental robustness, increase throughput and minimize manual labor [24,25].

## 3. Increased Cell Growth Rate

The microalgae are a group of prospective resources. Their cultivation is simple, and requires less fresh water and fertile land compared to the other terrestrial plants [26]. Microalgae can be used in the pharmaceuticals, nutraceuticals, biofertilizers, bioplastics, biofuels, cosmetics and feeds for aquaculture and poultry [27,28,29], consequently addressing environment pollution [30]. The productivity and yield are still the key indicators for the biotechnological and economic feasibility of microalgae [7]. Recently, genetic engineering is applied to improve microalgal growth for large-scale production, which can eventually realize the commercial utilization of microalgae [31,32]. However, the use of transgenic microalgae in outdoor aquaculture systems is still limited for security reasons. Therefore, increasing ideas focus on ALE training, which can avoid risks with high growth rate of microalgae. The existing stress methods for increasing cell growth rate are shown in Table 1.

Recently, the method to improve the growth rate is mainly performed through controlling light factors by using ALE. In the early process of exploring natural selection, to improve the performance of strains, *C. reinhardtii* was evolved for 1880 generations in liquid medium under continuous light. At the end of experiment, evolved cells had a growth rate that was 35% higher than the progenitor population [38]. This significant growth enhancement was largely due to the improvement of acetic acid metabolism, which showed that the utilization route of organic carbon in algae can provide direction for strain improvement. The process completely transformed the strain at the gene level. In other words, continuous light can enhance the utilization of acetic acid for fundamental processes, such as DNA replication and protein translation. Although whether the genetic modification was stable or permanent at that time was a subject for debate, it has clearly demonstrated the ability of algae genomes to adapt to environmental changes, and the potential of this strategy for future microalgae engineering, which is later known as ALE. Since then, there have been many studies on regulating light to increase cell growth rates. *C. reinhardtii* was cultivated in TAP media with a light intensity of 50 μmol photons m^−2^ s^−1^, the final biomass concentration can reach 1.48 times of the starting strains [39].

Light-emitting diode (LED) is a novel light source, which has the advantages of high efficiency, reliability, long life and low power consumption [40]. For microalgal cultivation, LED allows for artificial control of the spectral output, light intensity and light frequency for light configuration [35]. Biological productivity and light capture efficiency are crucial indicators to evaluate the economic feasibility of production mode by LED technology. Although LED is a little expensive, LED-based PBRs will become practical for producing algal biomass. Using LED-based PBRs for *C. vulgaris* can provide a biomass productivity of up to 2.11 g L^−1^ d^−1^, with a light yield of 0.81 g DCW/Einstein. This demonstrated that LED-based PBRs, combined with microalgae biotechnology, can efficiently convert carbon dioxide into biomass and valuable products. 

## 4. Improved Product Yield

Microalgae provide an abundance of value-added products that can accumulate up to 10–70% of specific biochemical substances (such as lipids and carbohydrates). These components have different functions because of various features. Carotenoids from microalgae can be used in medicine, cosmetics and food; lipid from microalgae can be used as a raw material to produce biodiesel to replace fossil fuels; carbohydrates produced by microalgae can be processed into bioethanol; and astaxanthin from microalgae has strong antioxidant activity and is used in health products, food and feed industries. However, the yield of these active products is not large enough to meet the industrial demand without external interference, so ALE can be applied to obtain high-yield strains for commercial utilization. The existing stress methods for improving product yield are shown in Table 2.

Because of the requirement to supply the global market, people are interested in extracting from algae and higher plants to gain β- carotenoids and lutein. Therefore, ALE has been applied to microalgae to produce a high content of carotenoids. It has been suggested that light plays a key role in the biosynthesis of carotenoids, through light signal sensing and downstream regulation [65]. It is well known that β-carotene can be overproduced in the marine microalga *Dunaliella salina*, in response to stressful light conditions [50]. Previous study explored the effect of red light-emitting diode (LED) lighting on growth rate and biomass yield, which identified the optimal photon flux for marine *D. salina* growth. The red-light photon stress alone at a high level was not capable of upregulating carotenoid accumulation. Therefore, combining red LED (75%) with blue LED (25%) allowed growth at a higher total photon flux, and increased β-carotene and lutein accumulation [50]. An efficient culture system with increased light energy efficiency and economy of operation can be developed in combination with genetically based methods, such as ALE for strain development.

*Haematococcus pluvialis* is considered as the best natural source to product astaxanthin. The antioxidant capacity of astaxanthin is the most outstanding than other carotenoids, which endows astaxanthin in suppressing tumor growth, improving body immunity and the scavenging of active oxygen and free radicals [66]. Many efforts are devoted to increase astaxanthin yield from *H. pluvialis*. More astaxanthin was obtained at the melatonin concentration of 5–15 mu mol/L, at 27–29 °C and light intensity of 198–216 μmol photons m^−2^ s^−1^ [67]. The application of nitrogen stress and excess light based on fed-batch culture can improve lipid and astaxanthin productivity to 457.1 and 2.0 mg L^−1^ d^−1^ [61]. The study revealed excess light can lead to more available carbon molecules to synthesize astaxanthin.

Microbial lipids often contain abundant polyunsaturated fatty acids, including eicosapenteanoic acid (EPA), DHA and arachidonic acid (AA), which can be used as the source of functional food and raw materials for green biofuels. ALE strategy has greatly improved the ability of microbial oil production, aiming to culture special microalgae strains with convenient, cost-saving and high-yield oil. The salt stress and nutrient osmotic pressure are widely used to increase lipid content in ALE [46].

These stressful conditions have largely changed the carbon flux to the expected products. ^13^C-MFA is used to be an accurate tool for describing the central carbon metabolism [68]. There is a conflict between lipid accumulation and cell growth; the massive accumulation of lipid is against for cell division. To resolve the conflict between cell growth and lipid accumulation, a general countermeasure is two-stage cultivation strategy. The first stage is for a rapid growth to gain the maximum biomass production, subsequently the second process for the lipid accumulation under various stress conditions at the expense of cell growth [17,69]. Studies have shown that *Parachlorella* sp. can be cultured in two stages, increasing the total FA productivity to 219.0 ± 10.7 mg L^−1^ d^−1^, and the biomass reached by 80% [29]. Similarly, in the first stage, microalgae grew under red LED to obtain the maximum biomass; in the second phase, green LED was used for stress to produce a large amount of lipids [70]. 

However, two-stage culture strategy can also lead to large labor expenditure and economic consumption, so it is necessary to culture algae strains that can grow rapidly under stress for a long time. Therefore, transcriptome analysis of strains produced by ALE can be carried out to obtain two different metabolic responses to stress and reveal the different gene expression patterns among the strains. The understanding of microalgal evolution under stress is beneficial for the development of the strains with rapid growth under stress [71]. Based on gene expression patterns of different metabolic reactions under stress, the ALE can be applied to all lipid-rich microalgae.

## 5. Enhanced Environmental Tolerance

ALE, to enhance the environmental tolerance of strains (Table 3), is profitable for the industrial production of microalgae. For example, cyanobacteria, a group of Gram-negative prokaryotes capable of performing oxygenic photosynthesis [72], is a promising cell factory to convert carbon dioxide into useful chemicals by autotrophic mode. Since toxicity produced is a key challenge, when cyanobacteria is the host for photosynthetic production of chemicals, tolerance of cyanobacteria to solvents is required to improve by ALE. *Synechococcus elongatus* PCC 11801 was cultured in ALE stage, and a new strain resistant to n-butanol and 2, 3-butanediol was obtained, which also showed high tolerance to other alcohols. The evolved strain had high tolerance without obvious growth lag phenomenon compared with original strain. The mutation mechanism was revealed by whole genome sequencing [73]. 

Light factors of light source, light quality, light intensity and light cycle affect microalgal growth. Microalgae have different appropriate light intensities. The light compensation points of microalgae can only maintain cell basic metabolism without growth. The higher light intensity promotes microalgal growth. However, when the light intensity reaches the saturation point of photosynthesis, microalgal growth rate no longer increases. A further increase of light intensity will lead microalgae to suffer a photoinhibition. At present, studies indicated that enhanced light tolerance of the cyanobacterium *Synechocystis* by ALE could increase the biomass. By combining repeated mutagenesis and exposure to increasing light intensities, the modified strains can grow rapidly under extremely high light intensities [75]. The ALE maximized photosynthesis and thus increased the accumulation of photosynthetic products, converting carbon dioxide into useful chemicals.

In addition, large-scale cultivation of microalgae is mostly used for environmental governance. The atmospheric CO_2_ levels reached the “dangerously high” levels of 400 ppm. One of the reasons for the large emission of greenhouse gases is the combustion of fuel in power plants. Efforts are underway to scrub CO_2_ from flue gas emitted from pulverized coal power plants using carbon sequestration and storage technology [83], which will make the production costly. Subsequently, microalgae are rapid converters of solar energy to biomass by assimilating atmospheric CO_2_ [84]. There has been demonstrated to use industrial flue for culturing microalgae [85], but flue gas supplementation at higher flow rates leads to the acidification of the growth medium, which typically cannot be tolerated by microalgae. Therefore, the microalgal tolerance is required to enhance to offset carbon emissions from fossil fuel combustion. A study showed that mixed biodiverse microalgal communities can be selected and adapted to tolerate growth in 100% flue gas from an unfiltered coal-fired power plant that contained 11% CO_2_ [80]. Cheng et al. have reported that the adaptive evolution against simulated flue gas containing 10% CO_2_, 200 ppm NOx and 100 ppm SOx can obtain a new strain, *Chlorella* sp. CV, which can tolerate simulated flue gas conditions and the maximum CO_2_ fixation rate was 1.2 g L^−1^ d ^−1^ [81]. It can be helpful to establish a new process for CO_2_ capture directly from industrial flue gas.

## 6. Promoted Nitrogen and Phosphorus Removal in Wastewater

Wastewater, resulting from various farming, domestic and industrial water operations, has been a key pollution of the environment for a long time in many countries or regions [86]. However, current methods for wastewater treatment have many problems, such as high energy consumption and cost, and heavy secondary pollution [87]. It is reported that some microalgae can utilize external organic carbon sources to remove nitrogen and phosphorus efficiently, and absorb heavy metals by functional groups on its cell surface [88,89,90]. Moreover, microalgae can utilize the nutrients from wastewater discharge to form beneficial products, including methane and hydrogen [22,91,92]. The application of removing nitrogen and phosphorus from wastewater by microalgae is developing rapidly [93]. The existing stress methods for increasing the ability of nitrogen and phosphorus removal in wastewater are shown in Table 4.

As mentioned above, light stress is used to improve the biomass and biodiesel production of microalgae [103]. Therefore, in wastewater treatment, light stress increases microalgal biomass to improve the removal rate of nitrogen and phosphate. Mahsa et al. have reported that the total biomass and protein concentrations of marine *Spirulina platensis* were observed under blue light, at around 100 μmol photons m^−2^ s^−1^ of 13.4 and 9.0 g L^−1^, respectively. However, the highest phosphate and ammonium removal were about 145 and 218 mg L^−1^ under purple light, at around 100 μmol photons m^−2^ s^−1^, respectively [97]. Results showed that light intensity and wavelength, combined with semi-batch cultivation, can be designed to achieve the highest biomass and production, as well as to maximize the removal of phosphorous and ammonium.

Obviously, when microalgae absorb nitrogen, phosphorus and heavy metals in wastewater, their biomass and by-product yield are correspondingly increased. Previous research was to develop large-scale production to produce oil-rich algal biomass from wastewater. Almost all nutrients in sewage were consumed, and microalgal biomass and oil content could reach 2.0 g^−1^ L^−1^ d^−1^ and 25.25% (*w/w*) [97]; *Chlorella* cultivated in pig manure wastewater could also obtain 0.23 g m^−2^ d^−1^ fatty acid production capacity [98]. In addition, bacteriostatic compounds against antibiotic-resistant bacteria have been discovered from microalgae during phytoremediation of swine wastewater [99]. Aigars et al. have reported phosphorus removal from municipal wastewater and the biomass were enhanced after four microalgal species were exposed to a phosphorus starvation medium, which indicated that the species, N/P molar ratio in the wastewater and P content of biomass could control the efficiency of phosphorus uptake [99]. *C. vulgaris* was continuously cultured for 15 days in municipal wastewater, in 3 temperature regimes. In this study, the analysis revealed that in alternating high–low temperature conditions, biomass production had the potential for biofuel production, with the highest lipid content (26.4% of total dry biomass) [95]. Therefore, some microalgae have the great potential to remove nutrient from wastewater and produce valuable compounds, simultaneously.

The technologies aim at promoting nitrogen and phosphorus metabolism in microalgae, by converting the nutrients into biomass [91]. There are two main pathways of nitrogen and phosphorus removal with microalgae for efficient wastewater treatment. One is performed through biochemical pathways for the uptake of nutrient components into the biomass for nucleic acids and proteins production. Additionally, the other can ingest phosphate for storing as an acid-insoluble polyphosphate granule [104].

Nowadays, microalgae technology is promising for wastewater treatment. However, adaptability metabolism of microalgae to extreme conditions in industrial wastewater is poorly understood. The microalgae harvesting in wastewater requires equipment investment and large amounts of energy, as the biomass concentration is usually below 0.6 g L^−1^ after the treatment. Therefore, efficient microalgal systems to treat wastewater at a large-scale production are in urgent needs.

## 7. Industrial Application of ALE in Microalgae

There are two mainly aspects about the industrial application of ALE in microalgae. Firstly, ALE is used as an optimal tool to improve the photosynthetic efficiency of microalgae in PBRs. The low and high light intensities limited the microalgal productivity at a large-scale production. ALE can develop an evolved microalgal strain with a new fixed trait, which can later be used as industrial strain for the enhanced production of the target metabolite. With the growing greenhouse effect, it is very exciting to produce biofuels, such as lipid, bioethanol, methane and hydrogen from microalgae via ALE.

Secondly, ALE has been applied to improve microalgal strains for industrial wastewater treatment. Since phosphorus and nitrogen in wastewater are the major nutrients causing eutrophication of aquatic ecosystems [99], it is necessary to search for a more effective means of nutrient removal. ALE can obtain microalgal strains for rapid nutrient loading, high tolerance to heavy metal ions and low nutrient effluent concentration [105]. At present, *Chlorella* sp. and *S. obliquus* are the most promising unicellular algae in wastewater treatment.

## 8. Challenges and Prospects

ALE is an emerging approach in artificial conditions, imitating the process of natural evolution to improve organisms, and being performed for various purposes at the laboratory level. The industrial microorganisms have become a key producer in various fields, including food, pharmaceuticals and other value-added chemical production [106]. ALE is one of the most effective approaches to eliminate obstacles and maximize productivity in bio-based processes, having advantages, such as the easy control of culture conditions, short generation time, easy manufacturing and storage of living fossil records for each period [107].

Meanwhile, based on these known influencing conditions, suitable combinations can make the effect more significant. Recent research showed that ALE can be combined with genetic engineering to improve the efficiency of gene transformation [108]. ALE was applied to streamline fitness recovery of genomically recoded *Escherichia coli*, for industrial-scale protein production [108]. High salinity accelerated the synthesis of phospholipids to restore lipid, and ALE culture was therefore carried out to obtain an evolutionary strain of *Nitzschia* sp. to increase lipid content to 51.2% [109]. Therefore, future research can combine several factors to obtain higher returns. 

However, ALE currently still faces many challenges. First is the formation of large mutant libraries and the need for large-scale screening of the required evolutionary bodies. Because the generation of genetic diversity has become a core technology for accelerating ALE, a high-quality mutant library is crucial to its success [6]. Second, with cell division, effective genes can disappear. The application of multi-omics analyses can promote the efficient data mining for the implementation of ALE experiments. The third is that the time span of ALE experiments is very long, and the end point is entirely up to the researcher’s decision. The novel cultivation strategy, such as red LED light and phytohormone, can potentially accelerate ALE progress based on microalgal cell physiology. Last, the strains produced by ALE have multiple mutations, and the interpretation of genotypes requires tedious omics analysis. The upgrade and development of related software will provide more effective data for ALE performance. 

Whatever the purpose, its essence is to continuously culture microalgae under certain pressure, screen out the most adaptable strains and constantly culture to meet our expectations. It is in response to “natural selection and survival of the fittest”. With in-depth research to conquer those difficulties, ALE combined with systems biology and synthetic biology tools is a prominent strategy. It is acknowledged that ALE is bound to become a popular technology in microalgae for the future.

## Figures and Tables

**Table 1:** Summary of targeting increased growth rate.

Stress Type	Strain	Stress Effect	Reference
Light intensity	*Chlamydomonas* *reinhardtii*, CC-124, CC-124H, CC-124 L	Fast growth rate cultivated on 120 μmol photons m^−2^ s^−1^	[33]
	*Microcoleus vaginatus*	The biomass can arrive in 546.0 mg L^−1^	[34]
	*Chlorella vulgaris*	Biomass density rose to approximately 20 g L^−1^ under 680 nm LEDs	[35]
Carbon	*Eubacterium limosum* ATCC 8486	Significant increased optical density (600 nm) and growth rate by 2.14 and 1.44 folds, respectively, under syngas conditions with 44% CO over 150 generations	[36]
	*Haematococcus pluvialis*	Biomass and astaxanthin yields t in an atmosphere comprising 15% CO_2_ were 1.3 times and 6 times higher than in normal air	[37]

**Table 2:** Summary of targeting increased product yield.

Stress Type	Strain		Stress Effect	Reference
Carbon	*Haematococcus pluvialis*		Oil content increased to 35.2% under 15% CO_2_	[41]
	*Crypthecodinium cohnii*		DHA-rich lipids accumulation in the strain can increase by 15.49% at 45 g L^−1^ glucose concentrations	[42]
	*Chlorella pyrenoidosa* G32		Starch content in the first few days under high glucose stress was eight times higher than that under low glucose stress	[43]
	*Zymomonas mobilis* ATCC ZW658		Maximum ethanol productivity attaches to 3.3 g L^−1^ h^−1^ in dual substrate mixture containing 5% (*w/v*) of glucose and 5% (*w/v*) xylose	[44]
Salt	*Synechocystis* sp. CCNM 2501		β-carotene produced at 1 M salinity is three times higher than the control	[45]
	Marine *Phaeodactylum tricornutum*		The addition of 20 g L^−1^ NaCl increased the total FA productivity to 219.0 ± 10.7 mg L^−1^ d^−1^, and the biological yield reached 80% of the salt-free culture	[46]
	Marine *Schizochytrium* sp.		Showed a maximal cell dry weight (CDW) of 134.5 g L^−1^ and lipid yield of 80.14 g L^−1^ under 30 g L^−1^ NaCl medium	[47]
	*Chlamydomonas reinhardtii*		Lipid content (73.4%) and lipid productivity (10.9 mg L^−1^ d^−1^)	[48]
	Marine *Dunaliella salina*		When salt concentration was increased from 4 to 9%, β-carotene yield was increased by 30-fold	[49]
Light	Light quality	Marine *Dunaliella salina*	The all-trans β-carotene and lutein content was increased to 3.3 times and 2.3 times of initial levels combining red LED (75%) with blue LED (25%)	[50]
		Marine *Stauroneis* sp.	The highest EPA proportions and yields were obtained under blue LED in f/2 medium (16.5% and 4.8 mg g^−1^) and the fucoxanthin yield was the highest when cells were subjected to blue LEDs (5.9 mg g^−1^)	[51]
	Light intensity	*Haematococcus pluvialis*	The highest astaxanthin accumulation with 15.76 mg g^−1^ in the experimental group with light intensity of 350 μmol photons m^−2^ s^−1^	[52]
		Marine *Phacodactylum tricornutum*	Biomass production and fucoxanthin accumulation enhanced under combined red and blue light	[53]
		Marine *Dunaliella salina*	The β-carotene production of 30 pg cell^−1^ d^−1^ under high light intensity	[54]
		*Desmodesmus* sp.	The light intensity resulted in an enhanced lutein productivity of 3.6 mg L^−1^ d^−1^	[55]
		*Haematococcus pluvialis*	Through a two-stage cultivation system in conjunction with light stress, a final astaxanthin productivity of 11.5 mg L^−1^ d^−1^ was obtained	[56]
Temperature	*Haematococcus pluvialis*		The net biomass and astaxanthin yields increased 5 and 2.9-fold under the culture temperature was 28 °C (daytime) and < 28 °C (night)	[57]
Oxygen	Marine *Schizochytrium* sp.		Observed 84.34 g/L of cell dry weight and 26.40 g L^−1^ of DHA yield with high oxygen	[58]
Nitrogen	*Chlamydomonas reinhardtii*		Total lipid content of the strain increased suddenly from 24.27% to 44.67% after nitrogen deficiency for 6 h	[39]
	*Synechococcus elongatus cscB*		The production of polyhydroxyalkanoates (PHA) of about 23.8 mg L^−1^ d^−1^ and a maximal titer of 156 mg L^−1^	[59]
	Marine *Synechococcus* sp. NKBG 15041c		Under nitrogen ambient (3 mM NaNO_3_) conditions also gave a higher yield of glycogen (404 μg mL^−1^ OD_730_^−1^)	[60]
	*Chromochloris zofingiensis*		Increase lipid and astaxanthin productivity to 457.1 and 2.0 mg L^−1^ d^−1^	[61]
Sulfur	*Chlamydomonas reinhardtii*		Lipid accumulation in sulfur-free medium was 66% higher than usual	[62]
Phosphorus	*Chlorella vulgaris*		Oil content in medium without KH_2_PO_4_ was 1.02 times higher than that in control group	[63]
Chemical regulator	*Crypthecodinium cohnii*		Adding sethoxydim to 60 μM doubles lipid production	[18]
Combined	Light and CO_2_	*Haematococcus pluvialismutant*	Yield of astaxanthin under 15% CO_2_ and strong light was 6 times higher than that of control group	[37]
	Temperatures and salinities	Marine *Schizochytrium* sp.	A maximal cell dry weight of 126.4 g L^−1^ and DHA yield of 38.12 g L^−1^ under concomitant low temperature and high salinity	[47]
	Light and nitrogen	*Limnothrix* sp. CACIAM25	Produced a high lipid content at a low level of NaNO_3_ concentration (1 g L^−1^) and a high level of light intensity (100 μmol photons m^−2^ s^−1^)	[64]

**Table 3:** Summary of targeting increased stress tolerance.

Tolerance Type	Strain	Stress Effect	Reference
Butanol	*Synechocystis* sp. PCC 6803	A 150% increase of the butanol (0.2–0.5% *v/v*) tolerance	[71]
	*Synechococcus elongatus* PCC 11801	A 100% improvement in concentrations tolerated (2–5 g L^−1^ n-butanol and 15–30 g L^−1^ 2,3-butanediol)	[73]
Temperature	*Symbiodinium* spp.	Tolerance to 31 °C	[74]
	Marine *Thalassiosira pseudonana* CCMP 1335	Tolerance to 32 °C	[75]
Light	*Synechocystis* sp. PCC 6803	Tolerance to 2000 μmol photons m^−2^ s^−1^	[76]
Cadmium	*Synechocystis* sp. PCC 6803	Tolerated CdSO_4_ with a concentration up to 9.0 µM	[77]
Acid	*Synechocystis* sp. PCC 6803	Tolerance to pH 5.5	[78]
Salt	*Chlorella* sp.	Tolerance to 30 g L^−1^ NaCl	[79]
Carbon dioxide	*Chlorella* sp.	They grew rapidly in 30% CO_2_	[80]
Oxygen	Marine *Schizochytrium* sp.	A 32.4% increase in dry weight	[58]
Flue gas	*Desmodesmus* spp.	Tolerance to 100% unfiltered flue gas	[81]
	*Chlorella* sp.	1.2 g L^−1^ d^−1^ CO_2_ fixation rate 2.7 g L^−1^ biomass concentration 68.4% carbohydrate content	[82]

**Table 4:** Summary examples of increasing the ability of nitrogen and phosphorus removal in wastewater ^1^.

Stress Type	Types of Wastewater	Strain	Removal Rate	Reference
Temperature	Municipal wastewater	*Chlorella vulgaris*	TN (96.5%) TP (99.2%) COD (83.0%) NH_3_-N (97.8%)	[94]
Light	Artificial wastewater	*Chlorella kessleri*	NO_3_^−^-N (88.1%)	[95]
	Municipal wastewater	Marine *Spirulina platensis*	PO_4_^3−^-P (93%) NH_4_^+^-N (83%)	[96]
Salt	Municipal wastewater	Marine *Dunaliella salina*	NO_3_^−^-N (100%) NH_4_^+^-N (75.5%) PO_4_^3−^-P (63.5%)	[97]
	Sludge liquor	*Chlorella vulgaris*	COD (85.3%) TN (99.6%)	[98]
Phosphorus	Municipal wastewater	*Chlorella vulgaris*	PO_4_^3−^-P (>99%)	[99]
		*Desmodesmus communis, Tetradesmus obliquus, Chlorella protothecoides*	DIP (>99.9%) DIP (>99.9%)	[100]
Sodium acetate	Municipal wastewater	*Scenedesmus obliquus*	TN (82.20%) TP (76.35%)	[101]
Phenol	Phenolic wastewater	*Chlorella* sp.	Phenol (100%)	[102]

^1^ TN: total nitrogen; TP: total phosphorus; COD: chemical oxygen demand; DIN: dissolved inorganic nitrogen and DIP: dissolved inorganic phosphorus.

## Data Availability

Not applicable.
